# Topical Application of JAK1/JAK2 Inhibitor Momelotinib Exhibits Significant Anti-Inflammatory Responses in DNCB-Induced Atopic Dermatitis Model Mice

**DOI:** 10.3390/ijms19123973

**Published:** 2018-12-10

**Authors:** Wenyu Jin, Wei Huang, Liqing Chen, Mingji Jin, Qiming Wang, Zhonggao Gao, Zhehu Jin

**Affiliations:** 1 Division of Dermatology, Department of Clinical Medicine, Medical College of Yanbian University, Yanji 133000, China; jinwenyu_13@163.com; 2 State Key Laboratory of Bioactive Substance and Function of Natural Medicines, Department of Pharmaceutics, Institute of Materia Medica, Chinese Academy of Medical Sciences and Peking Union Medical College, Beijing 100050, China; huangwei@imm.ac.cn (W.H.); chenliqing@imm.an.cn (L.C.); jinmingji@imm.ac.cn (M.J.); wqmxinyou@imm.ac.cn (Q.W.)

**Keywords:** momelotinib, atopic dermatitis, cytokine, DNCB, dendritic cell, JAK/STAT pathway

## Abstract

Atopic dermatitis (AD) is a chronic recurrent skin disease dominated by T-helper 2 inflammation. Momelotinib (MMB) is a novel JAK1/JAK2 inhibitor suppressing the signal transduction of multiple pro-inflammatory cytokines. Recent studies indicated that JAK inhibitor could play a therapeutic role in AD disease. In this study, we evaluated the efficacy of MMB as a novel JAK1/JAK2 inhibitor in DNCB-induced AD mice and TSLP-activated dendritic cells. Our data showed that topical application of MMB reduced the skin severity scores and total serum IgE levels, and alleviated the histological indexes including epidermal thickness measurement and mast cell number. Also, it was demonstrated that MMB down-regulated the mRNA expression of IL-4, IL-5, IFN-γ and TSLP, and inhibited the phosphorylation of STAT1, STAT3 and STAT5 in skin lesions. Moreover, MMB reduced the expression of CD80, CD86, MHCII and mRNA of OX40L in TSLP-activated dendritic cells. In general, our study suggests that MMB can improve the symptoms of AD and topical application of MMB can become a promising new therapy strategy for AD.

## 1. Introduction

Atopic dermatitis (AD) is a chronic recurrent skin disease characterized by complex interactions between activated T-helper 2 (Th2) lymphocytes, eosinophils, and mast cells [1]. In the past 30 years, the morbidity of AD in children was approximately 15% to 30%, while it was approximately 2% to 10% in adults. In industrialized countries, the morbidity of AD remarkably increased by 2 to 3 times [2]. The clinical manifestations of AD include facial and extensor eczema in infants and children and flexural eczema in adults, and often accompany with obvious itching, which seriously affect patients living quality. The main clinical treatment agents for AD are corticosteroids and calcineurin inhibitors (e.g., Tacrolimus), which could block antigen processing by binding to specific cell receptors and inhibiting the release of pro-inflammatory cytokines, and inhibit the T lymphocyte activated by Langerhans cells and production of inflammatory cytokines respectively. But the therapeutic efficacy in patients with moderate to severe AD is not ideal, and faced with a series of limitations and potential risks [3,4,5]. In recent years, with the in-depth exploration of association between AD and cytokines, biological agents targeting cytokines have been used in the treatment of AD. Except dupilumab (human mAb targeting interleukin (IL)-4 receptor α), the clinical efficacies of other biological agents are not satisfactory [6]. Therefore, targeting intracellular signal transduction pathways of cytokines may be a new treatment strategy of AD.

Janus kinase/signal transducers and transcriptional activator pathways (JAK/STAT) can mediate classical signaling for many cytokines and growth factors. After JAK phosphorylation, activated STATs can be transferred into the nucleus and regulate several target genes [7]. The JAK family includes JAK1, JAK2, JAK3, and Tyk2, while the STAT family comprises STAT1, STAT2, STAT3, STAT4, STAT5A/B, and STAT6 [8]. In immune and allergic diseases, JAK inhibitors interfered with the secretion and role of cytokines by blocking the JAK/STAT signaling pathway, and achieved satisfactory therapeutic effects [9]. In the past few years, the JAK3 inhibitor Tofacitinib has shown remarkable therapeutic effect in the treatment of AD [10]. However, compared with JAK3 inhibitor, the latest clinical research suggested that JAK1 selective inhibitors may have better safety and efficacy with less leukocyte decline [11]. Momelotinib (MMB) is an ATP-competitive inhibitor of JAK1/JAK2 and exhibits about 10-fold high selectivity versus JAK3, and now there is only the oral dosage form for the treatment of blood system diseases [12]. However, there is a lack of research report about the therapeutic efficacy for AD treatment with topical use of MMB so far.

As a novel cytokine, thymic stromal lymphopoietin (TSLP) participate in AD through affecting dendritic cells (DCs) and innate immune cells [13]. Epithelial cells of AD can secrete large amounts of TSLP after stimulation by allergens [14]. TSLP promotes DCs maturation and activates OX40L (OX40 ligand) on its surface. In addition, OX40L binds to OX40 on surfaces of the original CD4+ cells to promote the differentiation of Th2 cells, which ultimately leads to Th2 type inflammatory response in AD [15,16]. In this study, we investigated the therapeutic effect of MMB with topical use for AD treatment in 2,4-dinitrochlorobenzene (DNCB) induced AD model mice, and explored its therapeutic mechanism in TSLP-stimulated DCs.

## 2. Results

### 2.1. The Macroscopical Effect of MMB on AD

**Figure 1:**
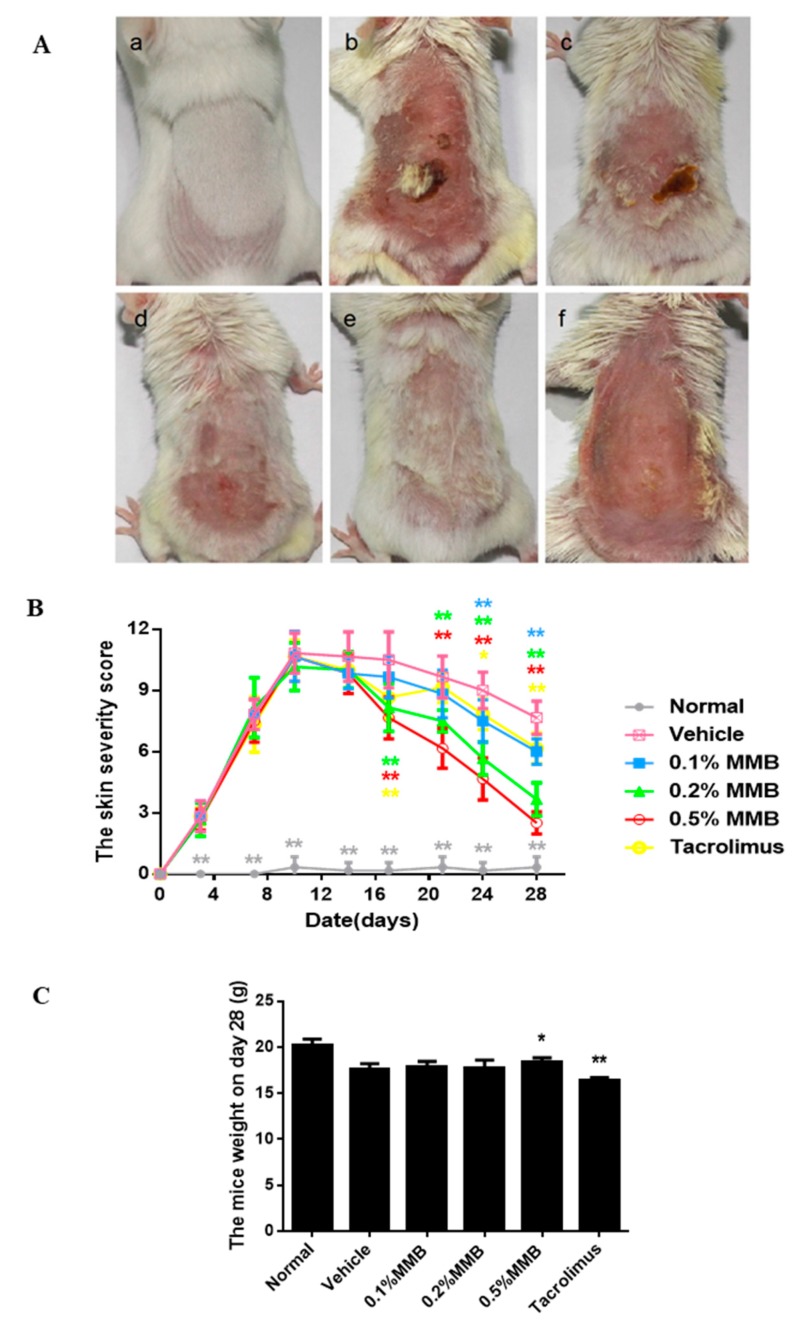
Momelotinib (MMB) ointment macroscopically alleviated the symptom of AD mice. (**A**) The skin lesion manifestation of groups on 28th day: (**a**) The normal group; (**b**) the vehicle group; (**c**) the 0.1% MMB group; (**d**) the 0.2% MMB group; (**e**) the 0.5% MMB group; and (**f**) the tacrolimus group. (**B**) The severity scores of skin lesion changes over time during the experiments. (**C**)The weight of mice on the 28th day of the experiment. Values are mean ± SD (*n* = 6). * p < 0.05, ** p < 0.01, versus the vehicle group.

Macroscopically, the back lesions of the vehicle group showed erythema, punctiform, hemorrhage, dryness, and crusting, a small amount of new hair, and the skin was significantly thickened. The symptoms of 0.1%, 0.2%, 0.5% MMB groups recovered to different extent (Figure 1A), especially in the 0.5% MMB group, which had no hemorrhage, crusting, and good hair condition. Meanwhile, the tacrolimus group showed significant erythema, dryness, and no newborn hair. The 0.1%, 0.2%, and 0.5% MMB ointment alleviated the symptom of the lesions and resulted in a decreased clinical skin severity score (Figure 1B) in a time- and dose-dependent manner. MMB started to decrease the skin severity score at day 17, and the score of 0.5% MMB group was lowest at day 28. The scores of 0.2%, 0.5% MMB, and tacrolimus groups decreased significantly from the 17th day (*p* < 0.001) and the 0.1% MMB group decreased significantly from the 24th day (*p* < 0.001), compared with vehicle group.

Meanwhile, we weighed the mice on the 28th day of the experiment (Figure 1C), and found that the 0.5% MMB group was significantly higher than that of the vehicle group (*p* < 0.05) while the tacrolimus group was significantly lower than that of the vehicle group (*p* < 0.01).

### 2.2. The Histological Effect of MMB on AD

**Figure 2:**
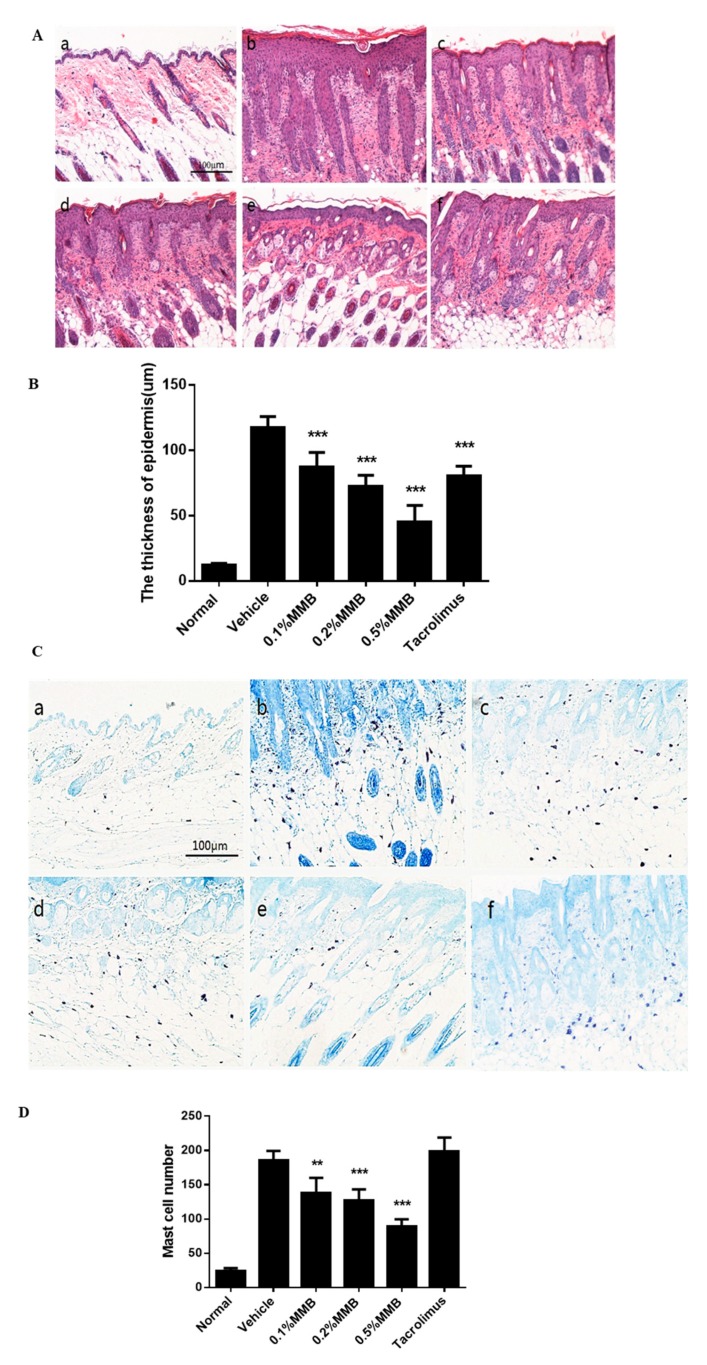
Skin lesions were stained with H&E and toluidine blue (×100 magnification, **A**,**C**) to represent the thickness of epidermis and number of mast cells respectively (×200 magnification, **B**,**D**). (**a**) The normal group, (**b**) the vehicle group, (**c**) the 0.1% MMB group, (**d**) the 0.2% MMB group, (**e**) the 0.5% MMB group, and (**f**) the tacrolimus group. Values are mean ± SD (*n* = 6). ** p < 0.01, *** p < 0.001 versus the vehicle group.

Hematoxylin–eosin (H&E) stains of skin lesion tissue showed that the reduction of epidermal thickness and inflammatory cells were observed in all treatment groups, most significantly in the 0.5% MMB group (Figure 2A,B). Toluidine blue stains of skin lesion tissue showed that reduced mast cells were observed in MMB groups. However, tacrolimus failed to inhibit the number of mast cells (Figure 2C,D). Above results revealed the topical application of MMB can improve the symptoms of AD histologically.

### 2.3. MMB Suppresses IgE Generation

**Figure 3:**
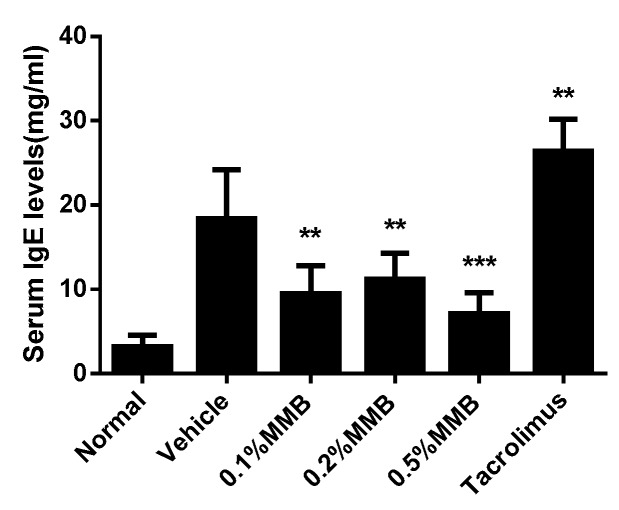
The total IgE levels in mice serum of different groups. Values are mean ± SD (*n* = 6). ** p < 0.01, *** p < 0.001 versus the vehicle group.

Total serum IgE was measured by ELISA. The result displayed that the IgE concentration in vehicle group increased by six times compared with normal group, showing obvious AD-like symptoms. Meanwhile, the MMB groups resulted in a marked decrease of serum IgE. However, no dose effect was shown. Unexpectedly, tacrolimus failed to inhibit the elevation of serum IgE levels. The reason may be that in hapten-induced AD model, tacrolimus cooperated with hapten to increase serum IgE, and this is also demonstrated by other studies [17,18] (Figure 3).

### 2.4. MMB Suppresses the Increased mRNA Expression of Cytokines in Skin Lesion

**Figure 4:**
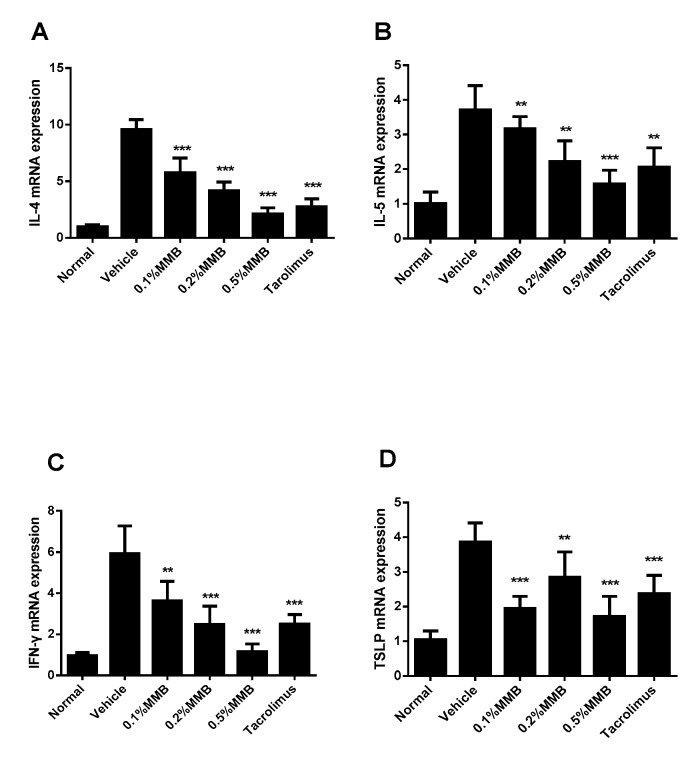
The relative mRNA expression of IL-4 (**A**), IL-5 (**B**), IFN-γ (**C**), and TSLP (**D**) in different groups. Values are mean ± SD (*n* = 6). ** p < 0.01, *** p < 0.001 versus the vehicle group.

The effect of MMB on the mRNA expression of cytokines in lesion tissues was detected by RT-qPCR in the work. The mRNA expression of IL-4, IL-5, and IFN-γ (Figure 4A–C) were increased in the vehicle group and decreased expression was observed in all MMB and tacrolimus groups in a dose-dependent manner. Furthermore, the mRNA expression of TSLP (Figure 4D) was notably decreased in all MMB and tacrolimus groups, compared with the vehicle group. However, no dose dependence was observed in MMB groups.

### 2.5. MMB Suppressed Expression of p-STAT1, p-STAT3, and p-STAT5 Proteins in Skin Lesions

**Figure 5:**
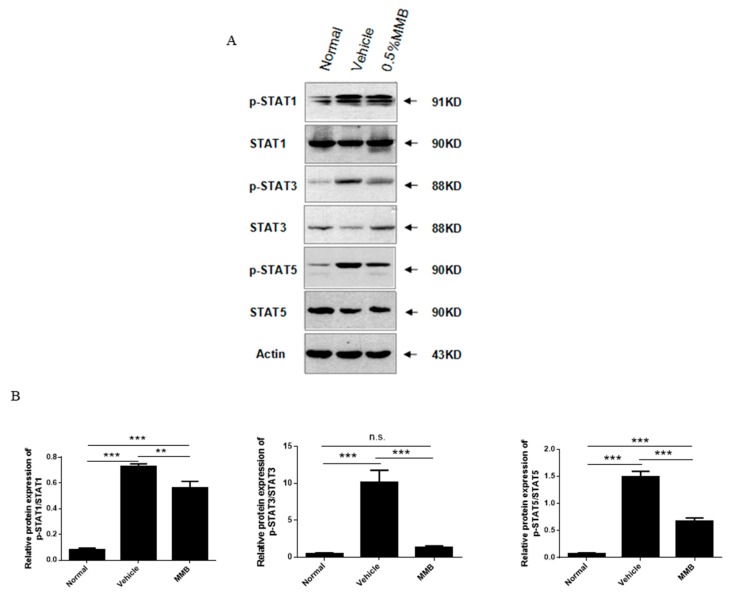
The protein levels of p-STAT1; p-STAT3; p-STAT5; and STAT-1, STAT-3, and STAT5 in normal, vehicle, and 0.5% MMB groups, respectively. (**A**) Western blot and (**B**) quantitative analysis of dot intensity. Actin was used as a reference protein. Values are mean ± SD (*n* = 3). ** p < 0.01, *** p < 0.001, n.s.: no statistical significance.

Western blot results exhibited that the expression of p-STAT1, p-STAT3, and p-STAT5 proteins were observably increased in the vehicle group. In the 0.5% MMB group, the expressions of p-STAT1, p-STAT3, and p-STAT5 were markedly inhibited (Figure 5). These results suggest that the JAK/STAT signaling pathway may be participated in the stimulation of DNCB-induced atopic dermatitis mice. Moreover, the effectiveness of MMB on AD mice may contribute to the down-regulation of p-STAT1, p-STAT3, and p-STAT5 proteins.

### 2.6. MMB Suppressed the Costimulatory Molecules, and Downmodulated the OX40L mRNA of TSLP-Stimulated DCs

**Figure 6:**
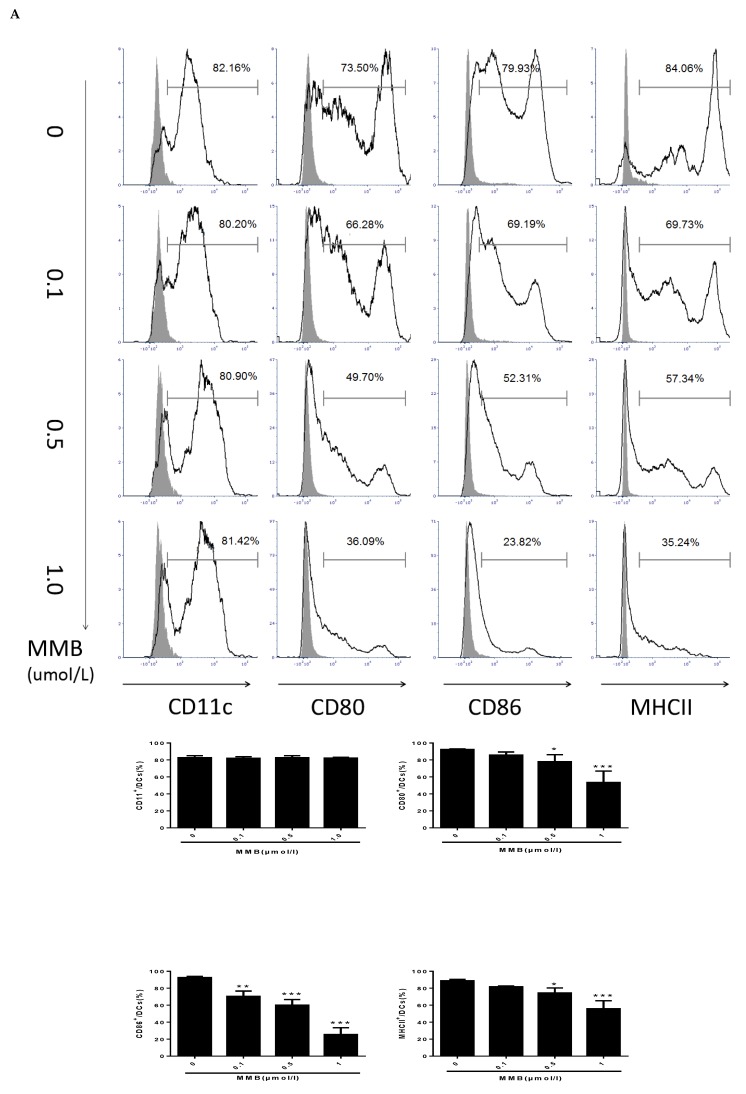

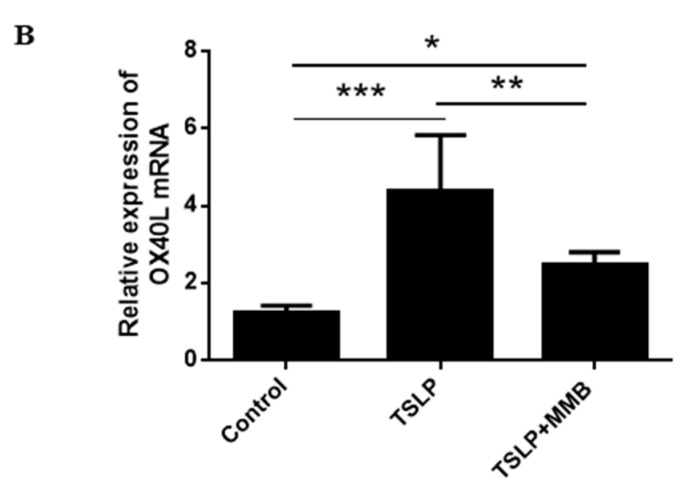
Effect of MMB on costimulatory molecules and OX40L in TSLP-induced dendritic cells (DCs). (**A**) The expression of CD11C, CD80, CD86, and MHCII. Values are mean ± SD (*n* = 3). * p < 0.05, ** p < 0.01, *** p < 0.001 versus 0 MMB group. (**B**) The relative mRNA expression of OX40L. Values are mean ± SD (*n* = 5). * p < 0.05, ** p < 0.01, *** p < 0.001.

To verify the suppressive effect of MMB, we used FACS to measure the costimulatory molecules of DCs, which were critical for DC function. Our data demonstrated that MMB showed a dose-dependent reduction of the expression of MHCII, CD80, and CD86, but no effect on CD11C (Figure 6A). Furthermore, the mRNA expression of OX40L was upregulated by TSLP in DCs. However, 0.5 µmol/L MMB reversed this influence (Figure 6B). In general, our results implied that blocking the JAK1/JAK2 might interfere the maturation of DCs and lead to a reduction of OX40L expression.

## 3. Discussion

The pathogenesis of AD is complex, and partly due to the imbalance of immune response mediated by JAK/STAT signaling. Th cells differentiate into Th1 and Th2 cells through the JAK/STAT pathway. Th1 cells differentiation require STAT1 to activate T-bet transcription factor, and T-bet is the main adjustment factor of Th1 cells differentiation. The differentiation of Th2 cells requires IL-4-stimulated STAT6, and STAT6 reversely activates GATA3 serving as the major regulator of Th2 cell development [19]. It has been proved that the activation of JAK/STAT pathway is also redox sensitive, and JAK inhibitors can be a good treatment agent for oxidative stress-related diseases [20]. Moreover, topical application of JAK inhibitor improved the function of skin barrier, and increased the expression of terminal differentiation proteins [21]. Therefore, targeting JAK/STAT can effectively regulate immune cell response, oxidative stress and skin barrier function, and played crucial roles in AD. Our study firstly demonstrated that topical application of JAK1/JAK2 inhibitor MMB obviously relieved AD symptoms in DNCB-induced AD mice. The symptoms of AD model mice in the treatment groups were improved in a time- and dose-dependent manner. The weight data also showed that the tolerance of AD mice to MMB ointment was better than that of tacrolimus. 

Histopathological sections of skin lesion treated with MMB also showed obvious reduction in the epidermal thickness and mast cells. Studies have shown that mast cells participate in the pathogenesis of AD by producing pro-inflammatory cytokines and chemokines. However, activated STAT3 and STAT5 are essential for mast cell function. Stem cell factor (SCF) binds with CD117 which is highly expressed on mast cells can induce the activation of JAK2 and then phosphorylate STAT5 and STAT6. IL-3, which can activate JAK2, STAT3, and STAT5, is necessary for regulating the immune response of mast cells [22]. Therefore, it is speculated that MMB can not only decrease the number of mast cells, but also regulate mast cell function by inhibiting the phosphorylation of STAT3, STAT5 in skin lesions.

The increase of total serum IgE is one of characteristics in immune environments of AD. After cross-linking with antigens, high level of IgE binds to FceRI on mast and basophils cells to initiate and amplify skin inflammation response [23]. Previous evidence suggests that STAT3 activation and B cell dysfunction may be the main cause of IgE level elevation in STAT3-mutated mice [24]. Our study showed that the serum IgE level in the MMB groups was obviously lower than that of vehicle group, and it was inferred that topical MMB reduced the level of serum IgE by inhibiting STAT3 phosphorylation.

The imbalance of Th1 and Th2 cells plays an vital role in the immune pathway of AD [25]. The expression of Th2 cytokines IL-4 and IL-5 in acute AD skin lesions were significantly increased than normal skin. IFN-γ, a Th1 cytokine, is highly expressed in the chronic AD skin lesion and positively correlated with the AD duration and severity of symptoms [26]. TSLP, a novel cytokine, may be a regulator in Th2-driven inflammatory diseases. Based on the above findings, we detected mRNA levels of these cytokines in skin lesions, and the results showed that the mRNA level of MMB groups were significantly lower than that of vehicle group. Additionally, IL-4 can promote the phosphorylation of JAK1 and JAK2 in keratinocytes, induce STAT-3 and STAT-6 nuclear metastasis, downregulate keratinocyte differentiation-related proteins and destroy the skin barrier [27]. IL-5 can also regulate the proliferation and survival of eosinophils through JAK2-STAT1/STAT5 and MAP kinase pathways [28]. IFN-γ can induce the expression of intercellular adhesion molecule-1 (ICAM-1) in human keratinocytes through JAK1/JAK2-STAT3 pathway [29]. TSLP can activate STAT5 in bone marrow cells, and the phosphorylated STAT5 can promote the proliferation of eosinophils and regulation of T cell proliferation and differentiation [30]. Therefore, we assumed that MMB improve AD symptoms not only by reducing cytokine expression, but also by inhibiting downstream pathways of cytokines.

Based on the above analysis, we speculated that topical MMB can alleviate symptoms and inflammation of AD lesion by improving histopathologic indexes as well as reducing related inflammatory cytokines and immune response. Meanwhile, the weights and Th1/Th2/Treg proportions of the lymph nodes and spleen were measured on the 28th day of the experiment. Compared with the vehicle group, MMB groups had no significant changes (data were not shown). So, it can be concluded that the topical use of MMB had no systemic effects on AD mice.

As antigen-presenting cells, DCs are widely found in skin, blood, bone marrow, and other organs that are closely related to allergic diseases, and participate in the initiating process of allergic reactions. TSLP is highly expressed in skin lesions of AD patients, and directly triggers DC-mediated allergic inflammation. In order to better understand the therapeutic mechanism of MMB, we co-cultured TSLP-stimulated BMDCs with MMB. The results exhibited that different concentrations of MMB (0.1, 0.5, 1µM) inhibited the expression of CD80, CD86, and MHCII on DCs in a dose-dependent manner. As we know, CD80 and CD86 support T cell activation, and MHCII is related to DC antigen presentation [31,32]. So, we inferred that MMB could adjust the functions of T cells and DCs through inhibiting the expression of costimulatory molecules on DCs.

TSLP-activated DCs promote naive CD4^+^ cells to produce the proallergic cytokines by up-regulating the expression of OX40L [33]. As a pair of costimulatory molecules, OX40/OX40L played a vital role in maintaining the proliferation and survival of CD4^+^ cells, the formation of memory T cells and the differentiation of Th2 cells. Increased expression of OX40L could enhance the Th2 polarization on CD4^+^ cells, resulting in increased number and function of Th2 cells [34]. So far, the operation mode understanding of TSLP signaling pathway has been a difficult problem in the research field of allergic diseases. Some studies suggested that TSLP stimulate DCs through JAK1/JAK2-STATs pathway in human, while TSLP directly activates STAT3 and STAT5 without JAK1/JAK2 in mice [35]. Thus, the roles of JAKs in this process still need to be further explored thoroughly. JAK phosphorylation is very difficult to detect in TSLP-induced DCs due to the rapidity of its phosphorylation process. Therefore, details of the JAK pathway have not been confirmed because there may be multiple cellular pathways involved in TSLP-induced DCs [36]. In this study, it was observed that the expression of OX40L was inhibited by JAK1/JAK2 inhibitor MMB, and it was demonstrated that MMB can exhibit a powerful therapeutic efficacy for AD through inhibiting the expression of OX40L and consequently adjusting the Th2 immune response. On the other hand, it was also suggested that JAK1/JAK2 participate in the process of TSLP-induced DCs in mice. But, the roles of JAK1 and JAK2 in this process still need to be further investigated in the future study.

## 4. Materials and Methods

### 4.1. Materials

MMB was purchased from BioChemPartner Co. Ltd (Shanghai, China). Tacrolimus (0.1%) ointment was provided by Astellas Pharma Inc. (Protopic^®^, Tokyo, Japan). DNCB was bought from Sigma-Aldrich (St. Louis, MO, USA). The IgE precoated ELISA kits were purchased from Dakewe (Shenzhen, China). Stat1; p-Stat1; Stat3; p-Stat3; Stat5; p-Stat5 were provided by Abcom (Cambridge, UK). Actin was purchased from CST (Boston, MA, USA). Polyvinylidene fluoride (PVDF) membrane was bought from Millipore (Billerica, MA, USA). TSLP was provided by USCN (Wuhan, Hubei, China); Trizol was bought from Invitrogen (Carlsbad, CA, USA). FITC anti-mouse CD11c; PE anti-mouse CD80; APC anti-mouse CD86; PerCP/Cy5.5 anti-mouse I-A/I-E; Cell Staining Buffer were provided by BioLegend (San Diego, CA, USA).

### 4.2. Animals

Female BALB/c and male C57BL/6 mice were obtained from Beijing Vital River Laboratory Animal Technology Co., Ltd (SCXK(Peking) 2016-0006). Animal care and all experiments were conducted ethically approved by the Principles of Laboratory. Animal Ethics Committee of the Institute of Materia Medica in Peking Union Medical College (Permit Number: 00005555; Approval Date: 12/03/2018).

### 4.3. Preparation of MMB Ointment

The preparation method of the vehicle is as follows: The oil phase: 2.5g of liquid paraffin, glycerol monostearate and white vaseline were mixed and heated to 80 °C. The aqueous phase: 2.5 mL Glycerol, 0.4 mL pan 80, 0.8 mL tween 80 were mixed and heated to 80 °C. Then the aqueous phase was gradually added to the oil phase and stirred until it condensed to form the ointment base. MMB (0, 10, 20, and 50 mg) was dissolved in dimethylsulfoxide (DMSO) and added to the above-mentioned base. Then water was added to above base until the total weight reached 10g and grinded in a mortar to obtain the vehicle, 0.1%, 0.2%, and 0.5% MMB ointments. For the positive control, 0.1% tacrolimus ointment was used.

### 4.4. Establishment and Topical Treatment of AD Model

36 BALB/c mice were randomly allocated into 6 groups (Normal; vehicle; 0.1% MMB; 0.2% MMB; 0.5% MMB; and tacrolimus). AD model (vehicle; 0.1% MMB; 0.2% MMB; 0.5% MMB; and tacrolimus) groups were established according to Lee et al [37]. Back hair of the mice was shaved a day before the experiment. For the sensitization process, an aseptic applicator with 100μl of 1% DNCB was attached to the shaved area on day 0 and 4. For the challenge process, the applicator with 100 μL of 0.2% DNCB attached to the sensitized area twice a week for a further 3 weeks.

**Figure 7:**
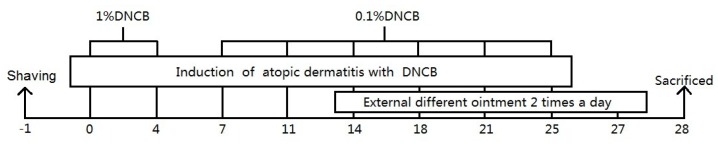
Schematic diagram of atopic dermatitis (AD) model establishment and treatment.

After the sensitization and challenge period of the first 2 weeks, MMB (0.1%, 0.2%, and 0.5%), tacrolimus, and vehicle ointment were applied topically to the back of each group twice per day for 14 days. On the 28 day, the eyeball blood and skin tissue of mice were collected for subsequent experiments (Figure 7).

### 4.5. Macroscopic Analysis

The scores of skin lesion severity were macroscopically assessed using previously established methods twice a week [37]. The totals scores of skin severity were evaluated for the following four symptoms (erythema/hemorrhage, edema, excoriation/erosion, and dryness) and defined as a sum of the individual score (0, no symptoms; 1, mild; 2, moderate; 3, severe). On the 28th day, we pictured the skin lesion and checked the weight.

### 4.6. Histopathological Analysis

The skin samples which embedded in paraffin were sectioned to 5 μm and then stained with H&E or toluidine blue. In the H&E staining section, histological changes were pictured (×100 magnification), and the thickness of epidermis were counted (×200 magnification). In the toluidine blue staining section the mast cells were pictured (×100 magnification), and the number in five random fields (×200 magnification) were counted under a biological microscope (DM4000B, Leica, Wetzlar, Germany).

### 4.7. ELISA Analysis

The blood samples were taken from the mice eyeball. After centrifugation, the supernatant was preserved at −80 °C. The IgE level were assessed using mouse IgE precoated ELISA kits according to the protocol.

### 4.8. Western Blot Analysis

50 mg of normal/vehicle/0.5% MMB group skin lesion tissues were used to extract protein. Protein quantification according to BCA method. Twenty micrograms of protein was loaded and separated on SDS ± PAGE (10%). After transferring to a PVDF membrane, the membrane was incubated overnight at 4 °C with antibody against p-STAT1 (1:5000), STAT1 (1:2000), p-STAT3 (1:1000), STAT3 (1:1000), p-STAT5 (1:1000), STAT5 (1:2000), or mouse monoclonal antibody against β-actin (1:1000). Five per cent BSA-TBST diluted secondary antibody and goat anti-mouse IgG HRP (1:10000) were incubated for 40 min at room temperature. Enhanced chemiluminescence (ECL) agent was added to the protein surface of the membrane for 3 min; film exposure: 10 s–5 min. The band density was analyzed with the IPP6.0 software (Media Cybernetics, Bethesda, MD, USA).

### 4.9. Preparation of DCs

DCs were isolated from femoral and tibial bone marrow cells of male C57BL/6 mice, as mentioned previously, with minor alterations [38]. Then, 1 × 10^7^ cells were cultured in 6-well plates in 4 mL bone-marrow derived dendritic cells (BMDC) medium, at 37 °C, in 5% CO_2_. On day 3, after gently pipetting the cells, the primary medium was replaced with fresh BMDC medium, and cultured for 2 days. Subsequently, 2 mL of BMDC medium was replaced and cultured until day 7. Immature DCs were collected and the surface costimulatory molecules were identified by flow cytometry. When CD11c^+^ cells had reached to 80% at day 7, DCs were stimulated with 150 ng/mL TSLP and different concentration of MMB (0, 0.1, 0.5, or 1 µM) simultaneously for 48 h.

### 4.10. Flow Cytometry

At day 9, the harvested cells were washed with PBS, resuspended in density of 1 × 10^6^ cells/mL. We then added the appropriate fluorescent concentration of anti-mouse dendritic cell surface molecules according to the instruction manual. CD11c-FITC, CD80-PE, I-A/I-E PerCP-Cy5.5, and CD86-APC were protected from light at 25 °C for 20 min. Stained cells were washed and analyzed by flow cytometry to detect CD11c, CD80 and CD86, and MHCII expression.

### 4.11. EvaGreen Real-Time Quantitative PCR

Total RNA was isolated using Trizol reagent, digesting DNA from sample RNA with DNase I. RNA reversed transcription to cDNA using M-MLV. Real-time qPCR was performed with 2× Ex TaqMix and EvaGreen dye, using a Roche LightCycler^®^ 480II under the following conditions: 95°C for 5 min, 95 °C 15 s→65 °C 30s (fluorescence detection), 45 cycles. The primers were as Table 1. The qPCR products were analyzed in triplicates. Results were normalized to the level of β-actin gene expression. Analysis of relative gene expression data was calculated using the 2^−^^ΔΔ^*^C^*^T^ method.

### 4.12. Statistical Analysis

All data were expressed as mean ± SD, statistical comparisons of different groups were performed using ANOVA. The simple effect analysis is based on the LSD method, and the α = 0.05 is the statistical benchmark. *p* < 0.05 was considered to be significant. All statistical tests were two-tailed. Statistical analysis and graphs were created using IBM SPSS 23.0 (SPSS Inc, Chicago, IL, USA) and GraphPad Prism 5.0 (San Diego, CA, USA).

## 5. Conclusions

In summary, we investigated the efficacy of MMB as a JAK1/JAK2 inhibitor in DNCB-induced AD mice and TSLP-activated dendritic cells. We demonstrated that topical application of MMB could reduce the skin severity scores and total serum IgE levels and alleviate the histological manifestation. Moreover, it was found that MMB could downregulate the expression of inflammatory cytokines including IL-4, IL-5, IFN-γ, and TSLP, which consequently reduced inflammatory responses in AD model mice. Surprisingly, topical administration of MMB has shown superior efficacy and fewer side effects than tacrolimus, and exhibited a great clinical value of further development. In further study, the comprehensive therapeutic effect of MMB in AD treatment still needs to be investigated, and also a better understanding of its role in the JAK/STAT pathway in AD will be explored.

## Figures and Tables

**Table 1:** Upstream and downstream primer sequences of genes.

Gene	Upstream Primer Sequences(5′-3′)	Downstream Primer Sequences(5′-3′)
*OX40L*	ACGCTAAGGCTGGTGGTCTCT	TCCTCACATCTGGTAACTGCTCCT
*IL-4*	CACGGATGCGACAAAAATCAC	CGAAAAGCCCGAAAGAGTCTCT
*IL-5*	TCCTCCTGCCTCCTCTTCCTGAA	TGTGATCCTCCTGCGTCCATCTG
*IFN-γ*	CCATCAGCAACAACATAAGCGTCA	CCGAATCAGCAGCGACTCCTT
*TSLP*	CTGCCATGATGAGGTGGTCTGAA	TCTGCTCACGAATTGTACTGTCCT
*β-actin*	GAGATTACTGCTCTGGCTCCTA	GGACTCATCGTACTCCTGCTTG
